# Application of a Microfluidic Gas-to-Liquid Interface for Extraction of Target Amphetamines and Precursors from Air Samples

**DOI:** 10.3390/mi11030315

**Published:** 2020-03-17

**Authors:** Michael Collins, Murat Gel, Chris Lennard, Val Spikmans, Shari Forbes, Alisha Anderson

**Affiliations:** 1 School of Science, Western Sydney University, Penrith 2751, Australia; c.lennard@westernsydney.edu.au (C.L.); v.spikmans@westernsydney.edu.au (V.S.); 2 CSIRO Manufacturing, Clayton VIC 3168, Australia; Murat.gel@csiro.au; 3 Département de chimie, biochimie et physique, Université du Québec à Trois-Rivières, 3351 boul. des Forges, Trois-Rivières, QC G8Z 4M3, Canada; Shari.Forbes@uqtr.ca; 4 Centre for Forensic Science, University of Technology Sydney, PO Box 123, Broadway NSW 2007, Australia; 5 CSIRO Health and Biosecurity, Canberra ACT 2601, Australia; Alisha.Anderson@csiro.au

**Keywords:** gas-to-liquid extraction, amphetamine, VOC, microfluidic, drug screening

## Abstract

The investigation of clandestine laboratories poses serious hazards for first responders, emergency services, investigators and the surrounding public due to the risk of exposure to volatile organic compounds (VOCs) used in the manufacture of illicit substances. A novel gas sampling interface using open microfluidic channels that enables the extraction of VOCs out of the gas phase and into a liquid, where it can be analysed by conventional detection systems, has recently been developed. This paper investigates the efficiency and effectiveness of such a gas-to-liquid (GTL) extraction system for the extraction of amphetamine-type substances (ATS) and their precursors from the vapour phase. The GTL interface was evaluated across a range of different ATS and their precursors (methamphetamine, dimethylamphetamine, N-formylmethamphetamine, benzaldehyde, phenyl-2-propanone, ephedrine and pseudoephedrine) at concentrations ranging between 10 and 32 mg m^−3^. These gas samples were produced by a gas generation system directly in Tedlar^®^ bags and gas canisters for controlled volume sampling. When using gas sampled from Tedlar^®^ bags, four of the seven compounds were able to be extracted by the GTL interface, with the majority of the VOCs having extraction yields between 0.005% and 4.5%, in line with the results from an initial study. When samples were taken from gas canisters, only benzaldehyde was able to be detected, with extraction efficiencies between 0.2% and 0.4%. A custom-built mount for the GTL interface helped to automate the extraction process, with the aim of increasing extraction efficiency or reducing variability. However, the extraction efficiency did not improve when using this accessory, but the procedure did become more efficient. The results from the study indicated that the GTL interface could be employed for the collection of gaseous ATS and incorporated into mobile detection systems for onsite collection and analysis of volatile compounds related to ATS manufacture.

## 1. Introduction

Over the 2015–2016 financial year, Australian law enforcement agencies identified 575 clandestine amphetamine-type substances (ATS) laboratories [1]. ATS manufacture (including 3,4-methylenedioxymethylamphetamine (MDMA)) and pseudoephedrine extraction facilities accounted for 63.6% of all clandestine laboratory detections [1]. The route of synthesis was found to vary by state, with the majority of New South Wales laboratories favouring the hypophosphorous method, Queensland laboratories favouring red phosphorus, and Western Australia laboratories generally using the Nazi–Birch method. The Australian Criminal Intelligence Commission (ACIC) report also found that 68.5% of all laboratories were located in residential areas [1].

In conjunction with a myriad of potential hazards associated with investigating clandestine laboratories, their common occurrence in residential areas poses a health risk to the general public, who may come into contact with toxic compounds and chemical waste associated with ATS synthesis [2,3]. Clandestine laboratory investigation is also becoming more challenging with operators in different states preferring different manufacturing methods, preventing investigators from using a single targeted approach to response, investigation, and remediation. The occurrence of different synthetic methods raises significant health concerns for investigators, and first responders as potential hazards may be unknown, with possible exposure to toxic volatile organic compounds (VOCs) [4]. In the synthesis of methamphetamine (MA) from ephedrine/pseudoephedrine via complex iodine/phosphorus methods, for example, iodine, acids, phosphine and various toxic by-products have been identified [5,6]. Vapour concentrations for MA, iodine and hydrochloric acid have been detected at ranges between 0.01 and 5 mg m^−3^ [7].

Given potential hazards at clandestine laboratories, there is a need for the portable contactless screening and identification of hazardous VOCs [8,9,10]. The onsite detection of hazardous VOCs can assist in hazard mitigation for investigators and the public, as well as providing rapid onsite forensic intelligence to help progress investigations. One of the most common air sampling techniques is solid-phase microextraction (SPME), which has been well-documented for the analysis of a wide range of ATS as well as other illicit compounds that may be present at clandestine laboratories, such as cannabinoids, cocaine and cathinones [11,12,13,14]. Investigators in New Zealand have developed a dynamic SPME method for onsite sample collection at former clandestine laboratory sites. By encasing an SPME fibre in a stainless-steel tube and applying a vacuum to one end, they developed a dynamic SPME technique that allows for large volumes of air to pass over the fibre’s adsorbent coating. The fibre was then taken back to the laboratory for conventional gas chromatography mass spectrometry (GCMS) analysis. By using this sampling and analysis methodology, they were able to detect airborne MA in 5–20 min in areas, where swabs showed concentrations greater than 0.04 μg m^−2^ [15]. Capillary microextraction (CME) has also been researched as a viable method of volatile ATS detection. The CME technique consisted of PDMS-coated glass filter strips inside a glass tube that were used dynamically to detect ATS vapour in areas, where the vapour concentration ranged between 0.4 and 4.2 μg m^−3^. The CME analysed by GCMS fitted with a thermal desorption unit produced good reproducibility and was found to be approximately 30 times more sensitive than the dynamic SPME method [16]. Similar in principle to SPME, scent transfer units have also been developed that combine a vacuum mounted behind an absorbent gauze pad that draws large volumes of air through the pad, which can then be stored, thermally desorbed and analysed by standard forensic techniques [17]. Field-portable MS systems have also been investigated for their possible applications in a range of tasks surrounding clandestine laboratory investigation. Using an atmospheric-pressure chemical-ionisation MS system and a selective target mass fragment filter, ions indicative of MA analysis were able to be covertly detected, when the MS system was mounted in the rear of a car [18]. Ion mobility spectroscopy (IMS) has also been used as a screening tool during clandestine laboratory remediation to assess the level of MA present in common building materials [19]. However, these methods of VOC analysis often rely on laboratory-based detection techniques. Additionally, many different detection methods require samples to be in the liquid phase for analysis. A method that extracts VOCs directly from a liquid would reduce preparation times, need for specialised equipment and allow VOCs to be analysed by a greater range of forensic instrumentation. A technique that is capable of onsite VOC collection and detection would allow law enforcement agencies to rapidly assess potentially hazardous conditions and aid investigators in analysing VOCs in laboratories. One potential method for onsite detection is the use of biosensors that are capable of detecting targets in the liquid phase, and thus, a system that collects VOCs from a liquid substrate could be a potential method of onsite analysis [20,21].

A microfluidic gas-to-liquid (GTL) interface that uses an open capillary bed as a method of VOC capture has recently been developed [22]. The GTL interface dispersed 30 μL of a liquid capture agent throughout a microfluidic channel network. The microfluidic network significantly increased the surface area of the liquid, so that, when a gas sample containing VOCs was pumped through the interface, there was a rapid exchange of VOCs from the gas phase to the liquid phase. The liquid could then be taken and analysed using conventional detection equipment that required solution-based samples. In an initial study to characterise the GTL interface and the factors affecting the extraction efficiency, allyl methyl sulfide and hexanal were extracted through the interface. It was found that the extraction efficiency varied significantly depending on VOC type, input amount and concentration. It was also found that decreasing the interface temperature to 15 °C during sampling and introducing a lipophilic binding agent such as α-cyclodextrin (α-CD) increased extraction efficiency for compounds with low water solubility. Under optimal conditions, the microfluidic GTL interface exhibited extraction efficiencies of 3.7% and 30% for allyl methyl sulfide and hexanal, respectively [22]. It is hypothesised that such a microfluidic device could be capable of extracting volatile ATS in gas samples. In this study, we examined the efficacy of using this GTL interface as a screening tool for the extraction of ATS and the related precursors from vapour-phase samples. If the GTL interface is found to be an effective method of volatile ATS extraction, it has the potential to be used for clandestine laboratory investigation. The GTL interface was characterised in terms of the presence/absence of the target compounds, extraction efficiency (percentage yield) and extraction reproducibility from laboratory-prepared gas samples.

## 2. Materials and Methods

### 2.1. Microfluidic GTL Interface

**Figure 1:**
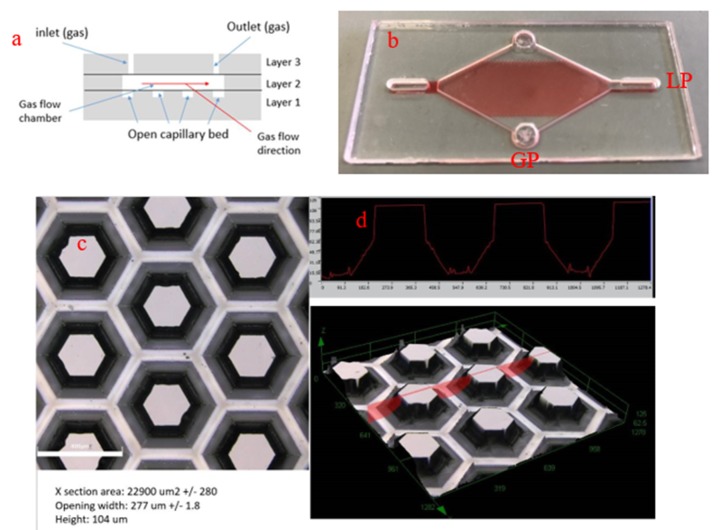
(**a**) Cross-section of a gas-to-liquid (GTL) interface showing 3 bonded layers. (**b**) Photo of the GTL interface with a solution of red food colouring dispersed throughout capillary channels, liquid ports (LPs) and gas ports (GPs). (**c**) Overhead image of hexagonal protrusions that form the capillary network. (**d**) Depth profile showing hexagonal protrusions beneath the red line (top) and 3D image (bottom) of the hexagonal protrusion network.

The GTL interface was a three-layered borosilicate glass chip that allowed for the rapid exchange of VOCs from the gas phase to the liquid phase [22] (Figure 1). The external dimensions of the interface were 65 mm × 30 mm × 3.3 mm. Layer 1 was the base of the GTL interface and incorporated a repeating tessellation of hexagonal protrusions with a size of 104 μm × 277 μm in a glass that formed an open capillary bed. These channels, when saturated, could hold 30 μL of a liquid capture medium and produce a gas contact area of approximately 3 cm^2^. Layer 2 was the gas chamber of the interface holding approximately 500 μL of gas. Layer 3 was the seal to the GTL interface and included liquid input and output wells for liquid priming and collection, in addition to two circular ports, which were approximately 4 mm in diameter, for introducing gas samples [22]. Gas extraction was achieved by pumping an air sample containing VOCs through the sealed microfluidic channel system via gas ports. The VOCs from the air sample were transferred into the liquid capture medium and, following extraction, were collected from the liquid output well using a syringe. 

### 2.2. GTL Extraction Mounts

A prototype mount was used for preliminary testing that optimised the extraction conditions identified during the initial characterisation study [22]. This mount consisted of a temperature controller connected to a thermal bed that the interface was inserted into for gas extraction. The thermal bed was set to 15 °C to work as a cold-trap, as this temperature was found to produce the highest extraction efficiency during the initial testing of the GTL interface. The prototype mount required manual flushing, cleaning, injection and collection of liquid from the interface, conducted using volumetric pipettes and gas-tight syringes.

**Figure 2:**
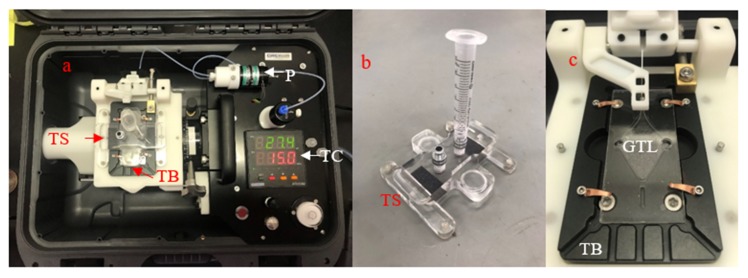
Grey Innovation GTL interface mount: (**a**) overhead view showing a thermal bed (TB), a transparent saddle (TS), a pump (P) and a temperature controller (TC); (**b**) transparent saddle with a syringe barrel attached; and (**c**) microfluidic GTL interface mounted on the thermal bed.

Grey Innovation (Melbourne, Australia) was tasked with designing an alternative interface mount that combined the functional elements of the prototype mount with the requirements for the pumping of gas and liquid through the interface. The developed product was a mount that replaced the manual flushing and priming of the microfluidic network with a pump, automating this part of the process (Figure 2). The thermal bed was also mounted on a camera pivot to maintain an appropriate angle during liquid collection at the end of the extraction process. The mount also replaced the sealing procedure used in the prototype with a saddle that sealed the interface shut with magnets, O-rings and septa. The saddle sat over the GTL interface and, when placed on top, sealed the liquid ports and replaced the septa used for sealing the gas ports with O-rings that led to luer locks, where syringe barrels could be attached (Figure 2b). The product was a mount that replaced the manual requirements of the prototype system with automated flushing, air-tight interface sealing and gravity feed systems. The Grey Innovation mount automated numerous manual handling steps, without intrinsically affecting the operation of the interface. The GTL interface priming, extraction and collection using the Grey Innovation mount are further outlined in Section 2.4. 

### 2.3. ATS Vapour Generation

Seven compounds were selected, as they represented common ATS on Australia’s drug market and the most common precursors, from which they are synthesised. All ATS and precursors employed in this study were purchased at 99% purity from the National Measurement Institute (West Lindfield, Australia). MA, N-formylmethamphetamine (FMA) and ephedrine were ordered as HCl salts that were subsequently converted to the free-base form and used to prepare 1000 ppm solutions in dichloromethane (DCM). Both dimethylamphetamine (DMA) and pseudoephedrine were purchased in the free-base form, and benzaldehyde and phenyl-2-propanone (P2P) were each prepared as 1000 ppm solutions in DCM.

To generate each ATS/precursor gas sample, an aliquot of the 1000 ppm solution in DCM was added to a 10 mL round-bottom flask (RBF), and the solvent was evaporated under a stream of nitrogen. The RBF was then sealed with a septum and heated in a bath of glass beads to the boiling point of each compound. When the glass-bead bath reached the boiling point, a nitrogen source regulated by a flow-controlled pump (SKC, Dorest, England) was pierced through the septum in the RBF via polytetrafluroethylene (PTFE) tubing fitted to a syringe. A 3 L Tedlar^®^ bag (AES, Auburn, Australia) filled with 2.5 L of nitrogen fitted with a syringe was also pierced through the septum in the RBF. The pump was positioned between the nitrogen source and the RBF to prevent pump contamination. Nitrogen at a flow rate of 50 mL/min was then pumped for 10 min (total 500 mL) from the source through the RBF containing the gaseous ATS/precursor to the Tedlar^®^ bag. The gaseous concentration in ppmV was determined using the following equation:

(1) $$\mathrm{ATS}=\frac{10^{6}\times L_{\mathrm{ATS}\mathrm{gas}}}{L_{\mathrm{total}}}$$

where $L_{\mathrm{ATS}\;\mathrm{gas}}\left(L\;\mathrm{of}\;\mathrm{ATS}\;\mathrm{as}\;a\;\mathrm{gas}\right)=n_{\mathrm{ATS}}\left(\mathrm{mol}\;\mathrm{of}\;\mathrm{ATS}\right)\times 22.4$ (molar volume constant).

When the vaporisation and the nitrogen dilution were complete, the Tedlar^®^ bag was connected to the GTL interface for microfluidic interface extraction.

An alternative method of gas generation and storage was also tested using gas canisters to determine if different methods of storage or gas collection would affect the measured efficiency of the GTL interface. These gas canisters were 6 L canisters designed for air analysis GCMS (AA-GCMS), with the interior walls having a silanol coating to prevent VOCs from binding to the surface. To prepare gas samples in the gas canisters, each target compound was vaporised in the same manner as above, but 10 mL of the gas from the RBF was drawn out using a syringe. This volume of the sample was injected into a tee-piece fitted with a septum under a flow of zero air fed to a canister, which was under vacuum. This resulted in the sample being drawn into the canister. The canister was then pressurised with zero air and left for 24 h to reach equilibrium. The concentration was determined using the following equation [23]:

(2) $$\mathrm{Cws}=\frac{\mathrm{Vss}\times \mathrm{Css}\times 14.7}{6\times P}$$

where Cws is the concentration of the working standard (ppbV), Vss is the volume of the stock standard injected (mL), Css is the concentration of the stock standard (ppmV), 14.7 is the value of 1 atmosphere pressure (psia), 6 is the canister volume (L), and P is the pressure (psia).

In order to regulate the flow from the canister through the GTL interface, gas was siphoned out into a Tedlar^®^ bag, where the flow could be controlled with the pump specified earlier. As the effectiveness of the GTL unit in extracting ATS from the vapour phase was unknown, high concentrations of ATS were used during testing.

### 2.4. Sampling and Vapour Extraction

**Figure 3:**
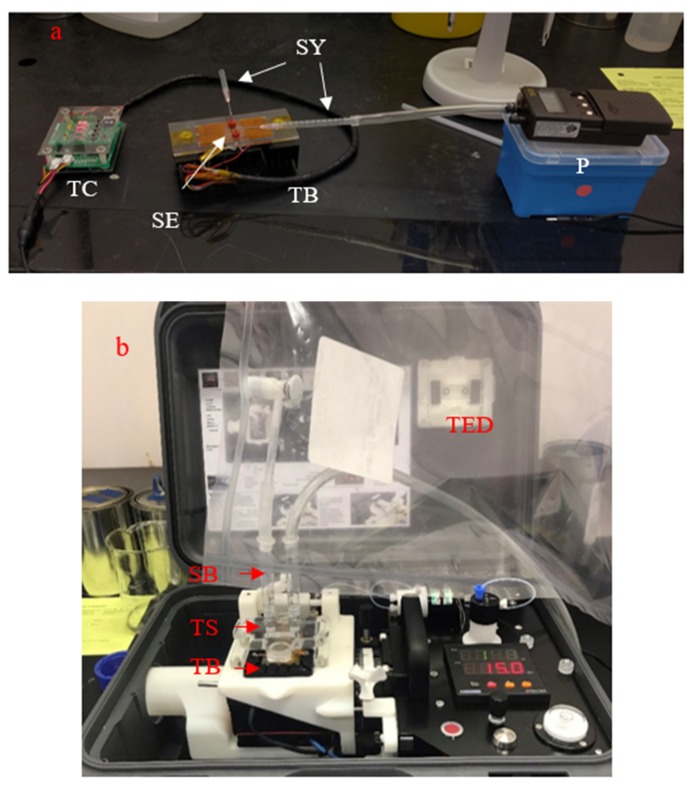
GTL extraction configuration of amphetamine-type substances (ATS) vapour: (**a**) prototype GTL mount showing the GTL interface in the thermal bed (TB) and fitted with septa (SE), syringes (SY), a pump (P) and a temperature controller (TC); and (**b**) Grey Innovation GTL mount showing the GTL Interface in the thermal bed (TB) fitted with a transparent saddle (TS), syringe barrels (SB) and a Tedlar^®^ bag (TED) containing a gas sample.

Prior to each use, the GTL interface was checked for water wettability by injecting 15 μL of an aqueous solution of red food colouring in Milli-Q water into microfluidic channels. If the dye ran smoothly through the microfluidic channels, then the channels had sufficient wettability. If the dye did not run smoothly, then the GTL interface was submerged in a 1 M solution of NaOH overnight to generate –OH groups on the glass surface to increase wettability [22]. To operate the GTL interface in the prototype mount, the GTL interface was placed on the thermal bed that was coated with a layer of oil to ensure good thermal contact. The temperature of the thermal bed was set to 15 °C. Septa were attached to the gas ports using a double-sided adhesive tape and further held in place by wrapping the tape around the GTL interface and the mount (Figure 3a). This was to ensure a gas-tight seal during extraction. Once mounted, the interface was cleaned by flushing with repeated 15 μL aliquots of Milli-Q water. It was then loaded with 3 × 15 μL aliquots of the 100 mM α-CD (Sigma-Aldrich, Castle Hill, Australia) solution in Milli-Q water. α-CD was used as a capture agent to assist in the extraction of highly hydrophobic compounds into an aqueous medium and showed a 10-fold improvement in extraction during the initial method development [22,24]. The mount was held at a 15° angle to gravity feed the solution to the liquid output port, where any excess solution could be collected and discarded, leaving 30 μL within the capillary bed. An excess volume of solution was used to ensure that the liquid in the capillary bed was solely composed of the α-CD solution and not the Milli-Q water from the flushing step. The liquid ports were sealed with a Kapton tape, and syringes were pierced through the septa into the gas inlet and outlet ports. The gas input syringe was connected to the Tedlar^®^ bag containing the target compound gas sample, and the output was connected to a pump that drew the gas from the bag and through the interface. The gas was extracted through the interface for 6 min at 50 mL/min (i.e., a total sampling volume of 300 mL). During extraction, the GTL interface and the mount were kept level to prevent the spillage of the extracted liquid. Following extraction, the gas input and output syringes and the tape were removed from the liquid ports, and 2 × 15 μL aliquots of Milli-Q water were injected into the liquid input port and the mount held at an angle to gravity feed the 30 μL of the α-CD solution, containing the extracted target compound, to the liquid output port, where it was collected with a gas-tight syringe and transferred to a GC vial. This extraction was repeated 7 times using the same Tedlar^®^ bag gas sample (2.1 L of gas was consumed in total, and the results were averaged). A bag containing pure nitrogen was also analysed as a control. The 30 μL extractions each underwent liquid–liquid extraction (LLE) by adding 100 μL of DCM and agitating for 1 min, after which excess sodium sulfate was added and agitated for 1 min to remove the water layer. The DCM layer was collected in a GCMS vial fitted with a 150 μL vial insert (Agilent, Mulgrave, Australia), and the extract was subjected to GCMS analysis. 

The extraction procedure using the Grey Innovation mount followed the process used for the prototype configuration but with automated steps replacing several manual handling steps in an attempt to improve the GTL interface efficiency and repeatability. The microfluidic GTL interface was placed on the thermal bed that was coated with a thin layer of oil, the interface was clamped down with small arms (Figure 2c), and the temperature was set to 15 °C. The same order of flushing and priming was then performed, but manual pipetting was replaced with an in-built pump that dispensed 15 μL of the solution at a time. Manually tilting the GTL interface was replaced with a camera pivot that the thermal bed was mounted on, so the same angle could be achieved for each extraction. Gas extraction occurred under identical conditions to those used for the prototype mount. Thirty millilitres of the α-CD solution containing the extracted target compound was collected in a similar manner, but the water used to flush out the capture solution was pumped in rather than by using a pipette. The LLE of each extraction aliquot remained unchanged (Figure 3b).

### 2.5. Instrument Parameters

The GCMS instrument parameters were based on the method used by the Forensic and Analytical Science Service (FASS, NSW Health Pathology, New South Wales, Australia) for the analysis of Schedule 8 drugs. Samples were analysed on a Shimadzu GC-2010 Plus Capillary GC fitted with a Shimadzu GCMS-QP2010 Ultra MS and a Shimadzu AOC-20i Auto Injector (Shimadzu, Kyoto, Japan). One microlitre of each sample was injected in the splitless mode into a 25 mm tapered liner (SGE Analytical, Ringwood, Australia). A Rxi-5ms (length × internal diameter × column: 30 m × 0.25 mm × 0.25 μm; Restek) was used for chromatographic separation with helium as a carrier gas set at a flow rate of 1 mL/min. Oven ramp conditions were as follows: initial temperature set at 50 °C held for 3 min, increased at a ramp rate of 10 °C/min to 200 °C and then at a ramp rate of 15 °C/min to 300 °C. The transfer line temperature was held at 260 °C. The ion source and quadrupole temperatures were set to 230 and 150 °C, respectively. The MS system was operated in the scan mode with a scan range (m/z) between 35 and 600 amu. Peak integration and identification were conducted using Shimadzu GCMSolution Version 4.20 and the NIST Mass Spectral Library (2008). Peak areas were determined via the extracted ion chromatograms for the base peak of each compound: benzaldehyde (106 m/z), P2P (43 m/z), ephedrine (58 m/z), pseudoephedrine (58 m/z), MA (58 m/z), DMA (72 m/z) and NFMA (86 m/z). In the case of ephedrine and pseudoephedrine, they were discriminated by comparison of retention times to standards. The concentrations of ATS were determined by taking the peak areas of the GTL aliquots and plotting them against their respective calibration curves. The mass yield was calculated by converting the calculated concentration (mg/L) into the mass (μg/100 μL) in the DCM extraction. The mass in the GTL aliquot was then divided by the calculated mass of ATS within the Tedlar^®^ bag to determine the yield (%) by mass.

## 3. Results

This study aimed to determine the efficiency and effectiveness of a microfluidic GTL interface for the extraction of ATS and precursors from gas samples to an aqueous solution that could then be analysed using traditional forensic techniques. Of the seven compounds tested in this research, four were able to be detected when prepared as vapour samples in a Tedlar^®^ bag and immediately extracted through the GTL interface (Table 1).

It can be seen from Table 1 that benzaldehyde and P2P had the greatest efficiency (defined as the mass of the compound collected in the extract divided by the calculated mass of the compound in the vapour sample), yielding 5.2% and 9.1%, respectively. MA and DMA behaved similarly, extracting 1.3%–1.5% of the ATS from the gas phase, and both displayed similar degrees of variance. The higher variance than those of all the other detected compounds is likely due to the lower vapour pressures of MA and DMA compared to those of the other three target compounds. FMA produced an extraction efficiency of <1% despite the similar structure and physical properties to those of MA and DMA. A duplicate Teldar^®^ bag was prepared with FMA and extracted, which confirmed the initial result as it produced a similar yield. The concertation of FMA in the GTL extract fell below the limit of detection (LOD), and although EIC exhibited visible peaks with the characteristic ions of FMA, its presence could not be confirmed or quantified with certainty. Viewing the data as a whole, none of the compounds displayed good repeatability when extracted through the GTL interface, producing relative standard deviation (RSD) values above 15%. The high variance among all compounds is likely a result of analysing low concentrations of nonvolatile compounds in the gas phase and difficulty with regimenting a vaporisation process that could be kept constant throughout the entire study. Ephedrine and pseudoephedrine were unable to be detected in any extraction. This finding is likely attributed to the extremely low vapour pressure of both ephedrine and pseudoephedrine, causing the VOCs to rapidly condense on any surfaces after vaporisation. A major limitation with this method of VOC vapour generation and extraction is that if a compound has a low vapour pressure then it will rapidly condense on cool (room temperature) surfaces post vaporisation, therefore resulting in negligible amounts in the gas phase for extraction through the GTL interface. It is important to note that the extraction efficiencies of the GTL interface were based off the assumed starting concentration in the bag, calculated from the volume of ATS initially used during sample vaporisation. In reality, the ATS concentration would likely be much less, as the ATS may not completely vaporise or may condense on any material such as tubing or bags that are at ambient temperature. The extraction yield of the GTL interface is therefore likely higher than those obtained.

Table 2 presents the data that were obtained when extractions were performed with the GTL interface fitted to the Grey Innovation mount. Two Teldar^®^ bags containing benzaldehyde and two bags containing P2P were prepared and extracted to determine if the automated aspects of this mount had any significant impact on the VOC extraction efficiency.

These results, when compared to those obtained using the prototype mount, showed that the Grey Innovation mount produced similar results for benzaldehyde. The benzaldehyde replicates were within a similar range as for the previous tests, given the high variance observed. In contrast with the previous results, the yield of P2P dropped significantly from 9.1% to 1.6%. The extraction efficiency of P2P when conducted using the Grey Innovation mount was more in line with the results seen for every other compound tested. Due to the extreme variance that was exhibited by all compounds, it was argued that the P2P result from the prototype mount producing a higher extraction efficiency was a result of the uncontrollable variables involved with sample vaporisation and that outlier results such as the P2P yield in Table 1 may not be uncommon. Repeated sample generation procedures using the same compound and volume generated under identical conditions may yield very different vapour concentrations within a Tedlar^®^ bag and hence different extraction efficiencies. Due to this reason, the method of sample vaporisation had a profound impact on extraction efficiency and if a method was used that could more precisely vaporise ATS, the variance exhibited by the GTL interface could more accurately be determined. Further studies are required across a wider range of compounds to determine if the Grey Innovation mount is beneficial in terms of the GTL extraction efficiency. As it stands, any potential positive or negative effect is being masked by the high variance exhibited by the method overall, regardless of which mount is employed. Aside from the analytical data, the Grey Innovation mount was found to improve the ergonomics of the extraction procedure by automating several manual handling steps. The new mount design also improved leak detection. This was due to the automation reducing the number of tubing connections and syringes. Moreover, fewer connections in this design needed to be checked to find the leak.

Combining both experiments, conducted on both the original prototype and Grey Innovation mounts, it can be seen that the GTL interface is capable of extracting target ATS and precursors from the gas phase to an aqueous solution for subsequent detection by GCMS. However, due to the high variance observed throughout the study, the results would not be suitable for quantification purposes. Four of the seven target compounds were able to be detected at low concentrations in small (300 mL) gas samples. This demonstrated that a GTL interface could be used as a screening tool for ATS and their precursors in the vapour phase.

### 3.1. GTL Interface Sampling in Gas Canisters

An alternative gas storage method was investigated using specialised gas canisters for air analysis to determine if it had an effect on the efficiency of extracting ATS using a microfluidic GTL interface. Of the seven compounds investigated, only benzaldehyde was able to be detected when prepared in a gas canister. A single canister was tested on two subsequent days to determine if similar extraction efficiencies could be obtained and to determine the sampling variance and variance between days (Table 3). Seven replicate extractions were conducted using the gas prepared in the canisters.

The results of the GTL extractions in the gas canisters indicated that using canisters as a gas preparation and storage vessel greatly reduced the perceived efficiency of the process from over 5% seen when prepared directly into a Tedlar^®^ bag to less than 1%. The concertation of benzaldehyde in the GTL extract fell below the LOD, and although EIC exhibited visible peaks with the characteristic ions of benzaldehyde, its presence could not be confirmed or quantified with certainty. Using a canister also had minimal effect on improving the repeatability of the extraction process given the high RSD observed across all tests. There are two possible explanations for this outcome. In the 24 h after the canister was left to reach equilibrium, the gas inside cooled. Low-volatility compounds could condense on the interior walls of the canister and be unavailable in the gas phase for extraction and analysis. Alternatively, elevated pressure within the canister may cause the vapour pressure of benzaldehyde to decrease, reducing the concentration of benzaldehyde in the gas phase. 

It is clear that Tedlar^®^ bags performed better than canisters in terms of the overall extraction efficiency. It is important to note, however, that the higher extraction efficiencies seen in the Tedlar^®^ bags are a reflection of the gas storage system, not the GTL interface itself. Different gas storage methods will change the concentration of the target compound in the gas sample that flows through the interface. As such, the calculated efficiency will change but not the actual interface efficiency. Therefore, it is important to investigate alternative gas storage methods. Evacuated gas canisters are an easy method to collect large volumes of gas without the need for pumps or polymer bags, and these are used routinely in air quality management. In addition to this, gas canisters also enable gas to be stored over long periods of time, whereas Tedlar^®^ bags should be used immediately as long storage times increase the risk of VOCs leaking through the pores in the bags or condensing on the walls of the bags. Canisters are also able to be cleaned and recycled, and hence they are cheaper compared to single-use Tedlar^®^ bags. There is still a place for the use of gas canisters for air sample collection at clandestine laboratory scenes; however, this would be restricted to more volatile ATS, precursors and solvents.

### 3.2. Methodology Limitations and Future Research

This research was conducted as part of the CYBERNOSE^®^ Project that is researching the application of next-generation chemosensory protein-based olfactory biosensors for the detection of VOCs [30]. Due to the overarching aims of the broader project, the methodology and assessment of a microfluidic GTL interface was limited in scope. It was a requirement to determine the extraction efficiencies for each of the seven compounds, of which the majority are semi- or nonvolatile. As such, analysing them in the gas phase had inherent limitations and resulted in a high degree of variation, which was expected and certainly observed. The choice of a liquid medium was also limited to water, despite the low solubility of the target compounds in this medium, as the GTL extractions are to be used in conjunction with biosensors that are only compatible with aqueous solutions. Compared with other VOC collection and detection studies such as the dynamic SPME method, the GTL interface required less gas, consuming only 300 mL, whereas the SPME method required 10 L. The GTL mass yields were comparable to those of the SPME method, which was able to detect ATS when their concentrations of >40 μg were present [15]. The CME method outperformed the GTL method and was more sensitive, but it did require larger volumes of gas [16]. The GTL interface using a liquid capture system does allow for the GTL extractions to be analysed by a broader range of forensic instrumentation. Although the extraction yields are low, emerging biosensor technology has the potential to be more sensitive than a GCMS system. If biosensors are sufficiently sensitive at these concentrations, then the low yields obtained may not be an issue. In addition, the aim of the project was to investigate the use of a GTL interface to screen for ATS and their precursors in the vapour phase. If the GTL interface can extract sufficient materials for biosensors to detect, then the GTL interface will work for its intended screening purpose. As this microfluidic technology is still in its infancy, many of the aspects that may affect extraction efficiency using a GTL interface are largely unknown. Factors such as capture agent composition and concentration, use of high flow pumps and extraction competition between different VOCs in the gas phase may have a large impact on extraction efficiency and need to be tested in depth. Further use of a GTL interface, focusing on more volatile precursors and reagents (including solvents) used in ATS manufacturing, may yield more successful results. In addition to this, the application of this device is not limited in scope to drug detection but also general air quality control, environmental management, food safety and other forensic applications such as explosives detection. We see this study as a starting point for the investigation of a broader range of potential applications.

## 4. Conclusions

A new microfluidic GTL interface was tested to determine its efficiency and effectiveness in extracting various ATS and their precursors out of the prepared vapour samples to a liquid medium (aqueous solution). The GTL interface was able to extract benzaldehyde, P2P, MA, DMA and FMA out of the prepared gas samples with a calculated 1%–10% extraction efficiency range, on average, based on an assumed starting concentration. These extractions were able to be conducted for 6 min using a 300 mL gas sample, showing that the microfluidic GTL interface has potential for use as a screening tool for the extraction of ATS and their precursors. However, due to the low volatility of the target compounds tested in this study, a relatively large concentration may need to be present in the vapour phase under ambient conditions to enable the extraction of a sufficient amount for traditional forensic analysis using GCMS or ultra-performance liquid chromatography (UPLC). This new microfluidic VOC capture technology is still in its infancy and requires further research to characterise its potential uses, operational range and limitations; however, this research provides a framework for further studies of this nature. With the level of customisation that is possible, including different capture agents, high-flow pumps and portable detection equipment, there is a myriad of potential air analysis applications. Provided that ATS and their precursors are present in sufficient concentrations in the gas phase under ambient conditions, the microfluidic GTL interface could provide a rapid, cheap, sensitive and robust alternative to conventional air sampling at clandestine laboratories 

## Figures and Tables

**Table 1:** GTL extraction of ATS and precursors using the prototype mount.

Target Compound	ATS as Vapour (μg)^1^	ATS Recovered (μg)	Concentration of GTL Extractions (μg mL^–1^)	Average Yield (%)	RSD of Replicates (%)	Vapour Pressure (mmHg at 25 °C) [25,26,27,28,29]	GCMS LOD (μg mL^–1^)
Benzaldehyde	100	5.2	52	5.2	31.4	1.2	5.05
Phenyl-2-propanone	40	3.6	36	9.1	26.2	0.2	2.4
Methamphetamine	140	1.9	19	1.3	45.6	0.005	3.1
Dimethamphetamine	140	2.1	21	1.5	42.4	Data Not available	5.5
N-formylmethamphetamine	120	0.05	0.5	0.04	15.8	Data Not available	3.1
Ephedrine	100	Not detected		-	-	0.001	6.9
Pseudoephedrine	100	Not detected		-	-	0.0008	1.8

^1^ Maximum amount theoretically possible in a 300 mL sampling volume of the four compounds that were detected in the GTL extract.

**Table 2:** GTL extraction of ATS and precursors using the Grey Innovation mount.

Compound	ATS as Vapour (μg)	ATS Recovered (μg)	Concentration of GTL Extractions (μg mL^–1^)	Yield (%)	RSD of Replicates (%)
Benzaldehyde (1)	100	2	20	2.0	28.0
Benzaldehyde (2)	100	4	40	3.7	48.9
Phenyl-2-propanone (1)	40	0.6	6	1.6	34.3
Phenyl-2-propanone (2)	40	0.6	6	1.6	25.4

**Table 3:** GTL extraction of benzaldehyde in gas canisters using the prototype mount.

Compound	ATS as Vapour (μg)	ATS Recovered (μg)	Concentration of GTL Extractions (μg mL^–1^)	Yield (%)	RSD of Replicates (%)
Benzaldehyde at day 1	30	0.3	3	0.1	28.6
Benzaldehyde at day 2	30	0.02	0.2	0.05	18.8
